# Recombinant Human Adenovirus-p53 Therapy for the Treatment of Cervical Cancer: A Meta-Analysis

**DOI:** 10.3389/fonc.2021.748681

**Published:** 2021-10-18

**Authors:** Yaru Guo, Jiuzhou Chen, Xiwen Zhang, Miao Fang, Mingna Xu, Longzhen Zhang, Enyu Rao, Yong Xin

**Affiliations:** ^1^ Department of Radiation, The Affiliated Hospital of Xuzhou Medical University, Xuzhou, China; ^2^ Cancer Institute, Xuzhou Medical University, Xuzhou, China

**Keywords:** cervical cancer, chemotherapy, radiotherapy, chemoradiotherapy, meta-analysis, recombinant human adenovirus-p53

## Abstract

**Objectives:**

To evaluate the clinical curative effects and toxicity of recombinant human adenovirus-p53 injection (rAd-p53) plus chemotherapy (CT), radiotherapy (RT), or concurrent chemoradiotherapy (CRT) for the treatment of cervical cancer.

**Methods:**

We identified 14 eligible studies in the PubMed, Web of Science, Cochrane Library, Embase, CNKI, Wangfangdate, CBM, and VIP databases from their inception to May 2021 and performed meta-analyses using RevMan version 5.3.

**Results:**

This analysis included 14 studies involving 737 patients. The results of the meta-analysis results showed significantly improved complete remission (odds ratio [OR] = 2.54, 95% confidence interval [CI]: 1.74–3.70, *p* < 0.00001), partial remission (OR = 1.56, 95% CI: 1.14–2.14, *p* = 0.006), and object response (OR = 4.47, 95% CI: 3.02–6.60, *p* < 0.00001) rates in the rAd-p53 combination therapy group compared to those in the CT/RT/CRT group. The results of subgroup analyses of CT/RT/CRT were consistent with the overall results. Regarding the incidence of adverse reactions, only the occurrence rate of fever (OR = 18.21, 95% CI: 10.54–31.47, *p* < 0.00001) in the rAd-p53 combination group was higher than that in the CT/RT/CRT group. No other significant differences were observed in other adverse reactions.

**Conclusion:**

RAd-p53 combined with CT/RT/CRT for the treatment of cervical cancer showed significant advantages in efficacy and safety compared to those in the CT/RT/CRT group. Therefore, rAd-p53 has great potential as an effective therapy for cervical cancer.

**Systematic Review Registration:**

https://inplasy.com/inplasy-2021-5-0058/.

## Introduction

Cervical cancer is the fourth most common malignancy in women worldwide and represents a major global health challenge. The Global Cancer Observatory reported approximately 570,000 cases of cervical cancer and 311,000 deaths from the disease in 2018, with approximately 290,000 (51%) of new cases worldwide occurring in women living in low- and middle-income countries (500,000 [88%], including upper-middle-income countries) (1–3).

Radical hysterectomy with pelvic lymphadenectomy remains the standard recommendation for patients with early-stage cervical cancer. The standard treatment for locally advanced cervical cancer is definitive chemoradiotherapy with pelvic radiotherapy (RT) and concurrent cisplatin-based chemotherapy (CT) (4, 5). However, many patients present at an advanced stage owing to the low cervical cancer screening rate (6). While concurrent chemoradiotherapy (CRT) is the standard treatment mode recommended by the National Comprehensive Cancer Network (NCCN) guidelines, 29%–38% of the failure modes are uncontrolled or recurrent disease, with a 5-year survival rate of relapsed patients of only 3.8%–13% (7). Chemotherapy and RT resistance are the major causes of cervical cancer recurrence and mortality (8, 9). Therefore, improving the sensitivity of RT and chemotherapy before treatment is particularly important to guide follow-up treatment (10).

The tumor suppressor gene p53 is widely regarded as the gene guardian of cells and plays a key role in cell cycle control, apoptosis, and the inhibition of tumor cell proliferation. The p53 gene is one of the most frequently mutated genes in human cancers, with over 50% of all cancers harboring p53 mutations (11). Inactivation of p53 function often correlates with increased malignancy, poor patient survival, and resistance to chemotherapy or RT (12–14). Accumulating evidence has demonstrated that the restoration of p53 activity can induce cell cycle arrest and apoptosis, eliminate RT and chemotherapy resistance, and inhibit tumor growth in cervical cancer cells (15). As such, the reactivation of the p53 protein has become an attractive approach for the effective treatment of cervical cancer (16). Recombinant human adenovirus p53 (rAd-p53) can transfer the p53 gene into tumor cells through recombinant human adenovirus-p53, rebuild p53 gene function in tumor cells, and cause tumor cells to undergo programmed death or develop a severe hibernation state, thereby increasing the sensitivity of tumor cells to RT and chemotherapy (17).

This meta-analysis aimed to systematically evaluate the efficacy and safety of rAd-p53 combined with CT/RT/CRT in the treatment of cervical cancer and to provide evidence-based medical data for the treatment of cervical cancer.

## Materials and Methods

This systematic review and meta-analysis was based on a pre-planned protocol constructed according to the standard Preferred Reporting Items for Systematic Reviews and Meta-Analysis (PRISMA) and was prospectively registered on inplasy.com (INPLASY protocol 202150058. doi: 10.37766/inplasy2021.5.0058).

### Study Inclusion Criteria

The inclusion criteria were as follows:

(i) Randomized controlled clinical studies.
(ii) Diagnosis of cervical cancer by cytological and histopathological examinations and without serious cardiac, pulmonary, hepatic, or renal disease.
(iii) Receipt of CT/RT/CRT combined with rAd-p53 for those in the experimental group was treated with and CT/RT/CRT alone for those in the control group.
(iv) The primary efficacy outcomes were complete remission (CR), partial remission (PR), and objective tumor response rate (ORR) according to World Health Organization (WHO) criteria. The ORR was calculated as follows: (CR + PR)/total number of cases×100%. The secondary outcome measure was the number of adverse reactions, including fever, myelosuppression, gastrointestinal reaction, radio rectitis, radio cystitis, and liver damage.

### Study Exclusion Criteria

(i) Non-randomized clinical controlled studies (RCTs), observational studies, and retrospective studies.
(ii) Duplicate studies and studies reporting incomplete or inconsistent outcomes.
(iii) Animal experiments, case reports, cohort studies, or review articles.
(iv) Included patients receiving other treatments in addition to rAd-p53 and CT/RT/CRT.

### Search Strategy and Study Selection

We identified RCTs of CT/RT/CRT plus rAd-p53 *versus* the control group without rAd-p53 in the treatment of cervical cancer through searches of the Cochrane Library, PubMed, Embase, Web of Science, Chinese National Knowledge Infrastructure (CNKI), Chinese Biological Medicine (CBM) Database, Wanfang Database, and the VIP Database until May 2021. We also searched for related trials in the International Clinical Trial Registry Platform (ICTRP) and the Chinese Clinical Registry. We used the following keywords along with medical subject heading (MeSH) terms: uterine cervical neoplasms, recombinant human adenovirus p53, chemotherapy, radiotherapy, and chemoradiotherapy. Two researchers independently screened the retrieved studies according to the inclusion and exclusion criteria set beforehand, with disagreements revolved by group discussion with a third researcher.

### Data Extraction and Quality Assessment

Two authors (YG and JC) independently extracted the relevant data, which included authors, year of publication, number of patients, age of patients, International Federation of Gynecology and Obstetrics (FIGO) stage, interventions, RT dose, and chemotherapy regimen. Two reviewers (XZ and MF) independently assessed the quality of the selected studies according to the Cochrane Collaboration tool for RCTs. The items were evaluated in three categories according to the risk of bias (low, unclear, and high risk of bias). The following characteristics were evaluated: random sequence generation (selection bias), allocation concealment (selection bias), blinding of participants and personnel (performance bias), incomplete outcome data (attrition bias), selective reporting (reporting bias), and other biases. The results were graphed and assessed using Review Manager 5.3.

### Statistical Analysis

We used Cochrane RevMan version 5.3 to analyze the data. The results were reported as pooled odds ratios (ORs) with respective 95% confidence intervals (95% CIs). Heterogeneity was assessed using Cochran’s *Q* test and *I* ^2^ statistics. If the heterogeneity was not significant (*p* > 0.1, *I* ^2^ < 50.0%), a fixed-effects model was used; otherwise, a random-effects model was used. The results of this meta-analysis were presented as forest plots. To detect potential publication bias, we generated a Begg’s funnel plot and performed a sensitivity analysis. All *p*-values were two-sided, and *p* < 0.05 was considered statistically significant.

## Results

### Study Characteristics

**Figure 1:**
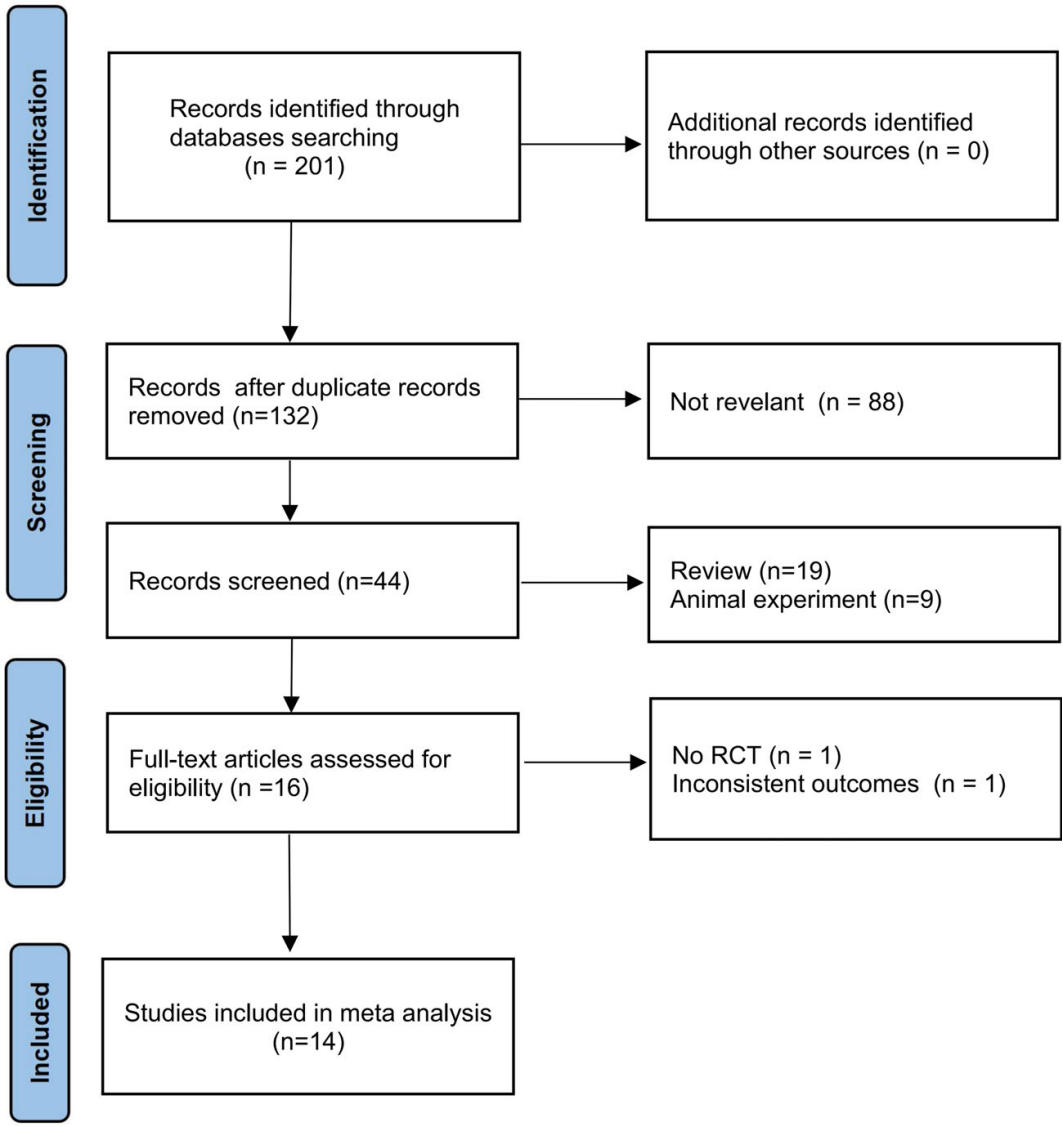
Flow chart of studies screening.

We identified 201 studies through the database search, 132 of which were duplicate studies, 88 were irrelevant research studies, 19 were reviews, and 9 were basic research based on title and abstract review. Among the remaining 16 articles selected for full-text review, we excluded one non-RCT and one article with an inconsistent outcome. Finally, the meta-analysis included 14 studies. A flow chart of the literature screening is shown in **Figure 1**. A total of 737 patients were enrolled, including 379 (51.4%) and 358 (48.6%) patients in the experimental and control groups, respectively. In the included literature, four RCTs (18–21) combined rAd-p53 with CT with platinum-based CT regimens; six RCTs (22–27) administered rAd-p53 with RT and four RCTs (28–31) combined rAd-p53 with CRT; among these 10 articles, six studies were rAd-p53 combined with intensity-modulated radiotherapy (IMRT), most commonly pelvic plus intracavitary RT. In the included literature, the experimental group was administered recombinant human P53 adenovirus injection-combined treatment based on the control group. Recombinant human P53 adenovirus injections (1 ×10^12^ virus particles) were removed from storage at −20°C and thawed at room temperature. The samples were then diluted in normal saline. Cervical tumors were injected once weekly for continuous treatment for 4–5 weeks. The detailed characteristics of each included article are summarized in **Table 1**.

**Table 1:** Characteristics of studies included.

Study	Study design	Sample size(Exp/Con)	Stage	Treatment		rAd-p53 dose	Radiotherapy		Chemotherapy
				Exp	Con		Radiotherapy types	Target area and radiation dose	
Xue YJ 2021 (18)	RCT	40/40	III–IV	rAd-p53 +CT	CT	1×10^12^VP time/week/4 weeks		–	Cisplatin: 50 mg/m^2^, d1-3 Paclitaxel: 80 mg/m^2^, d1, 8, 15
Jie X 2019 (20)	RCT	20/30	Ib2–IIa2	rAd-p53 +CT	CT	1×10^12^VP time/day/3 days		–	Cisplatin: 50 mg/m^2^, d1-3 Paclitaxel: 175 mg/m^2^
Zhang D 2019 (19)	RCT	40/40	IIIb–IV	rAd-p53 +CT	CT	1×10^12^VP time/week/8 weeks		–	Cisplatin: 25 mg/m^2^, d1-3, Paclitaxel: 80 mg/m^2^, d1, 8, 15
Cui L 2017 (28)	RCT	24/23	IIb–IIIb	rAd-p53 +CRT	CRT	1-2×10^12^VP time/week/2-4 weeks	IMRT	Pelvic radiotherapy: 50 Gy Intracavitary radiotherapy: 36 Gy	Cisplatin: 30 mg/m^2^/week/5-6 weeks
Zhang DJ 2017 (22)	RCT	21/25	II–III	rAd-p53 +RT	RT	1×10^12^VP time/week/2-4 weeks	IMRT	Pelvic radiotherapy: 50 Gy Intracavitary radiotherapy: 30–36 Gy	–
Jie X 2017 (21)	RCT	20/20	Ib2–IIIa	rAd-p53 +CT	CT	1×10^12^VP time/day/3days		–	Cisplatin+vincristine+bleomycin
Wang YJ 2017 (23)	RCT	40/40	IIa–IIIb	rAd-p53 +RT	RT	1×10^12^VP time/week/4-5 weeks	IMRT	Primary tumor and pelvic lymph node drainage area: 50 Gy Intracavitary radiotherapy: 24-36 Gy	–
Guo CA 2016 (29)	RCT	23/24	IIa–IIIa	rAd-p53 +CRT	CRT	1-2×10^12^VP time/week/5 weeks	IMRT	Cervix and tumors, all uterine bodies, iliac vascular lymphatic drainage area: 50 Gy	Cisplatin:25 mg/m^2^/ week/7weeks
Xing S 2016 (24)	RCT	69/35	IIb–IIIb	rAd-p53 +RT	RT	1×10^12^VP time/week/6 weeks	Conventional radiotherapy	Pelvic radiotherapy: 45 Gy Intracavitary radiotherapy: 20-30 Gy	–
Guo CA 2015 (30)	RCT	21/22	IIa–IIIb	rAd-p53 +CRT	CRT	1-2×10^12^VP time/week/4 weeks	Conformal radiotherapy	Cervix, uterine, body, and parauterine tissues and pelvic iliac lymphatic group: 50 Gy	Carboplatin: 130 mg 5-fluorouracil: 400 mg
Xu ZZ 2015 (25)	RCT	10/10	IIb–III	rAd-p53 +RT	RT	1×10^12^VP time/week/4-5 weeks	IMRT	Pelvic radiotherapy: 50.4 Gy Intracavitary radiotherapy: 24-36 Gy	–
Qian YQ 2013 (26)	RCT	20/20	IIa–IIIb	rAd-p53 +RT	RT	1-2×10^12^VP time/week/4-6 weeks	Conventional radiotherapy	Pelvic radiotherapy: 50 Gy Intracavitary Radiotherapy: 36 Gy	–
Qian L 2012 (31)	RCT	10/11	IIb–IV	rAd-p53+CRT	CRT	1×10^12^VP time/week/2-3 weeks	IMRT	Pelvic radiotherapy: 50-54 Gy Intracavitary radiotherapy: 30-36 Gy	Cisplatin: 50 mg/m^2^, d1-3 5-fluoroura: 325 mg/m^2^, d1-5
J pan 2011 (27)	RCT	21/18	IIb–IVa	rAd-p53 +RT	RT	14×10^12^VP time/week/6 weeks	Conventional radiotherapy	Pelvic radiotherapy: 45-50 Gy Intracavitary radiotherapy: 42 Gy	–

### Quality Assessment

**Figure 2:**
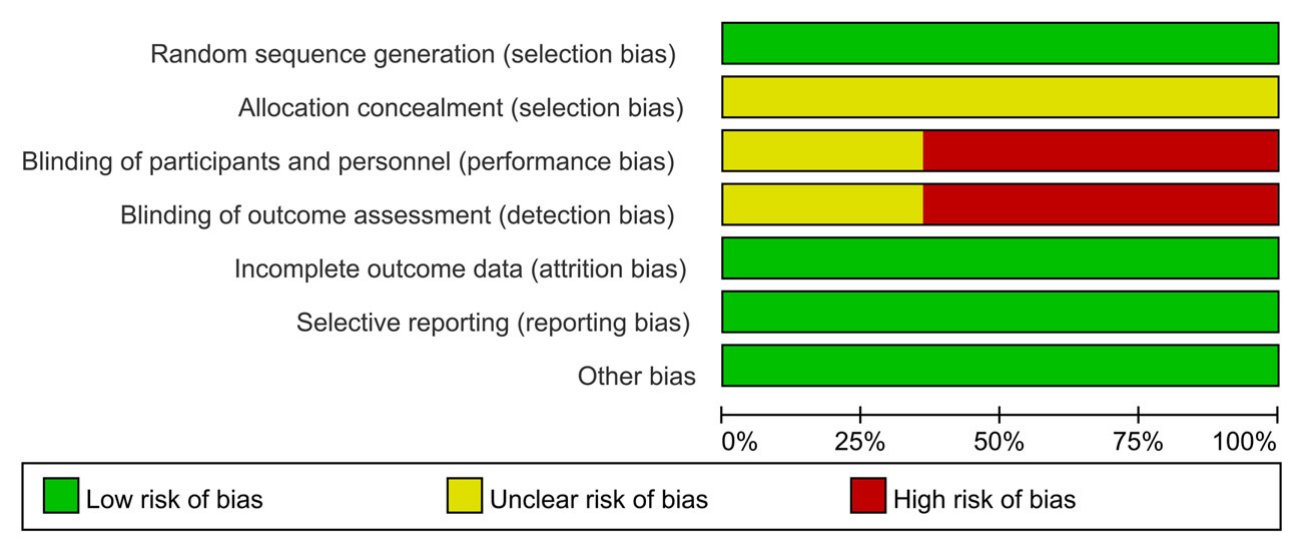
Risk of bias graph.

**Figure 3:**
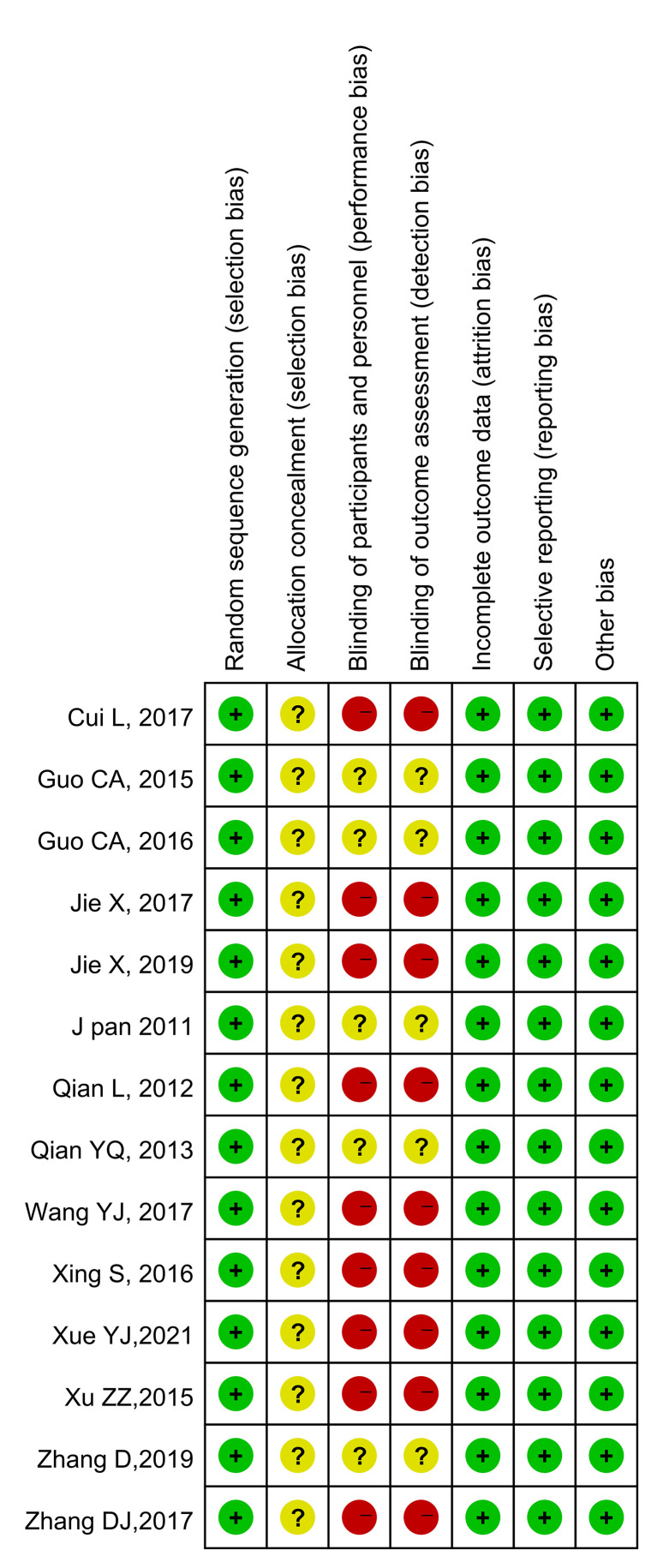
Risk of bias summary. Green indicates low risk; red indicates high risk; yellow indicates unknown risk.

The results of the quality evaluations of the included studies are shown in **Figures 2** and **3**. All the included studies were RCTs. Four studies assigned random numbers (18, 19, 24, 31), while none of the remaining studies described any particular randomization method. Most of the included studies did not provide sufficient information to assess whether the allocation concealment was adequate. A total of 488 participants in nine studies (18, 20–25, 28, 31) signed informed consent forms, while the remaining five studies (19, 26, 27, 29, 30) did not have sufficient information to assess the blinding method. All included articles had complete data, no data fall-off, no selective reports, and no other deviations.

### Efficiency

#### CR

**Figure 4:**
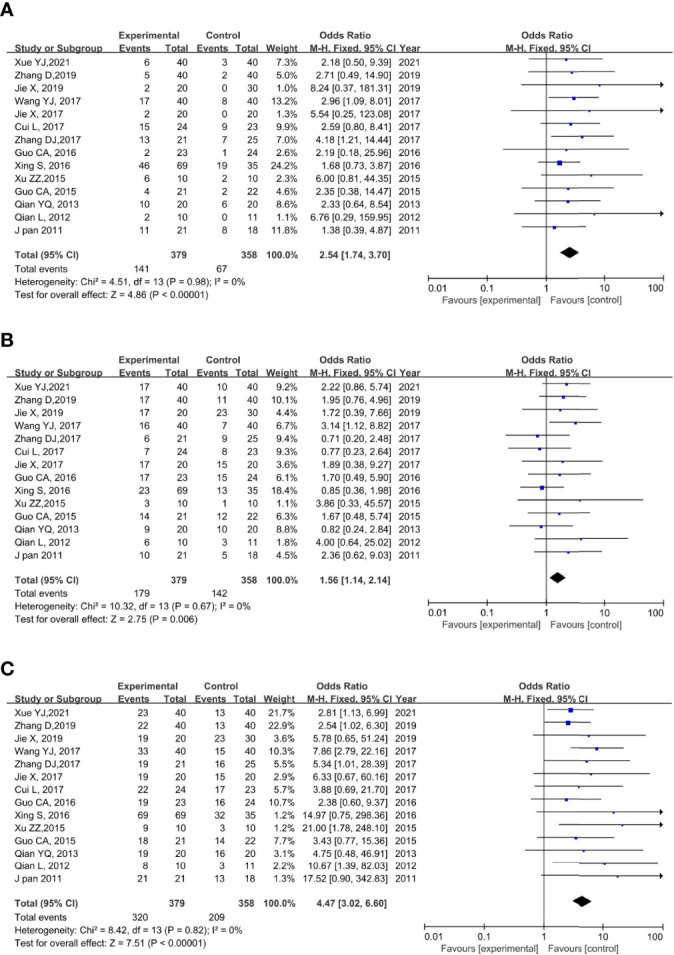
Forest plot for CR **(A)**, PR **(B)**, and ORR **(C)** of rAd-p53 combined CT/RT/CRT group and CT/RT/CRT alone group. CR: complete remission; PR, partial remission; ORR, objective tumor response rate; CT, chemotherapy; RT, radiotherapy; CRT, chemoradiation therapy.

All the included studies reported CR. None of the trials showed significant heterogeneity (*p* = 0.79, *I* ^2^ = 0%); thus, a fixed-effects model was used for the meta-analysis. The results of the meta-analysis showed a significantly higher CR rate in the rAd-p53 combination group compared to that in the control group (OR = 2.54, 95% CI:1.74–3.70, *p* < 0.00001) (**Figure 4A**).

#### PR

All 14 studies reported PR. We observed no statistically significant heterogeneity among the trials (*p* = 0.67, *I* ^2^ = 0%); thus, a fixed-effects model was used to perform the meta-analysis. The results of the meta-analysis showed a significantly higher PR rate in the rAd-p53 combination group compared to that in the control group (OR = 1.56, 95% CI: 1.14–2.14, *p* = 0.006) (**Figure 4B**).

#### ORR

All 14 studies reported the ORR. We observed no statistically significant heterogeneity among the trials (*p* = 0.82, *I* ^2^ = 0%); thus, a fixed-effects model was used for the meta-analysis. The results of the meta-analysis showed a significantly higher CR rate in the rAd-p53 combination group compared to that in the control group (OR = 4.47, 95% CI: 3.02–6.60, *p* < 0.00001) (**Figure 4C**).

### Subgroup Analysis

**Figure 5:**
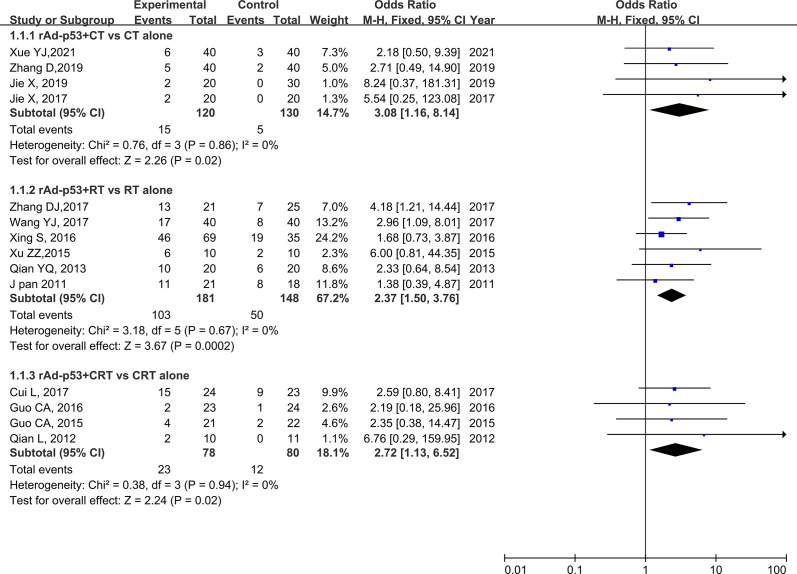
Forest plot for subgroup analysis of CR based on CT/RT/CRT. CR: complete. CT: chemotherapy; RT: radiotherapy; CRT: chemoradiation therapy.

**Figure 6:**
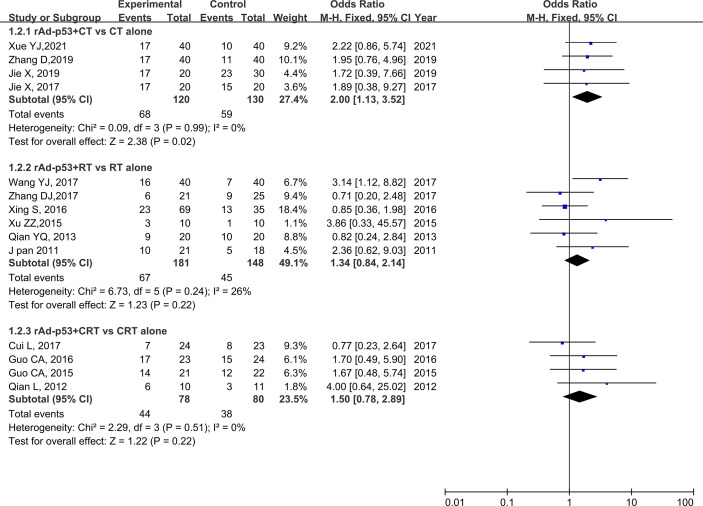
Forest plot for subgroup analysis of PR based on CT/RT/CRT. PR, partial remission; CT, chemotherapy; RT, radiotherapy; CRT, chemoradiation therapy.

**Figure 7:**
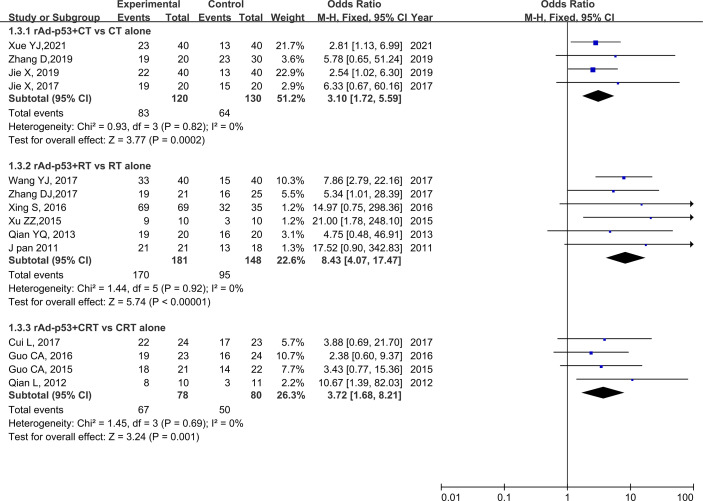
Forest plot for subgroup analysis of ORR based on CT/RT/CRT. CT, chemotherapy; RT, radiotherapy; CRT, chemoradiation therapy.

The subgroup analysis of CT/RT/CRT revealed no heterogeneity in any subgroup. The RT subgroup included four studies involving 250 patients; the RT subgroup included six studies involving 329 patients; and the CRT subgroup included four studies, involving 158 patients. All subgroups demonstrated improved CR, PR, and ORR (**Figures 5**–**7**). We also performed a subgroup analysis on IMRT/non-IMRT and observed no heterogeneity in all subgroups. Moreover, all subgroups demonstrated improved CR/PR/ORR (**Supplementary Figure S4**).

### Adverse Effects

**Figure 8:**
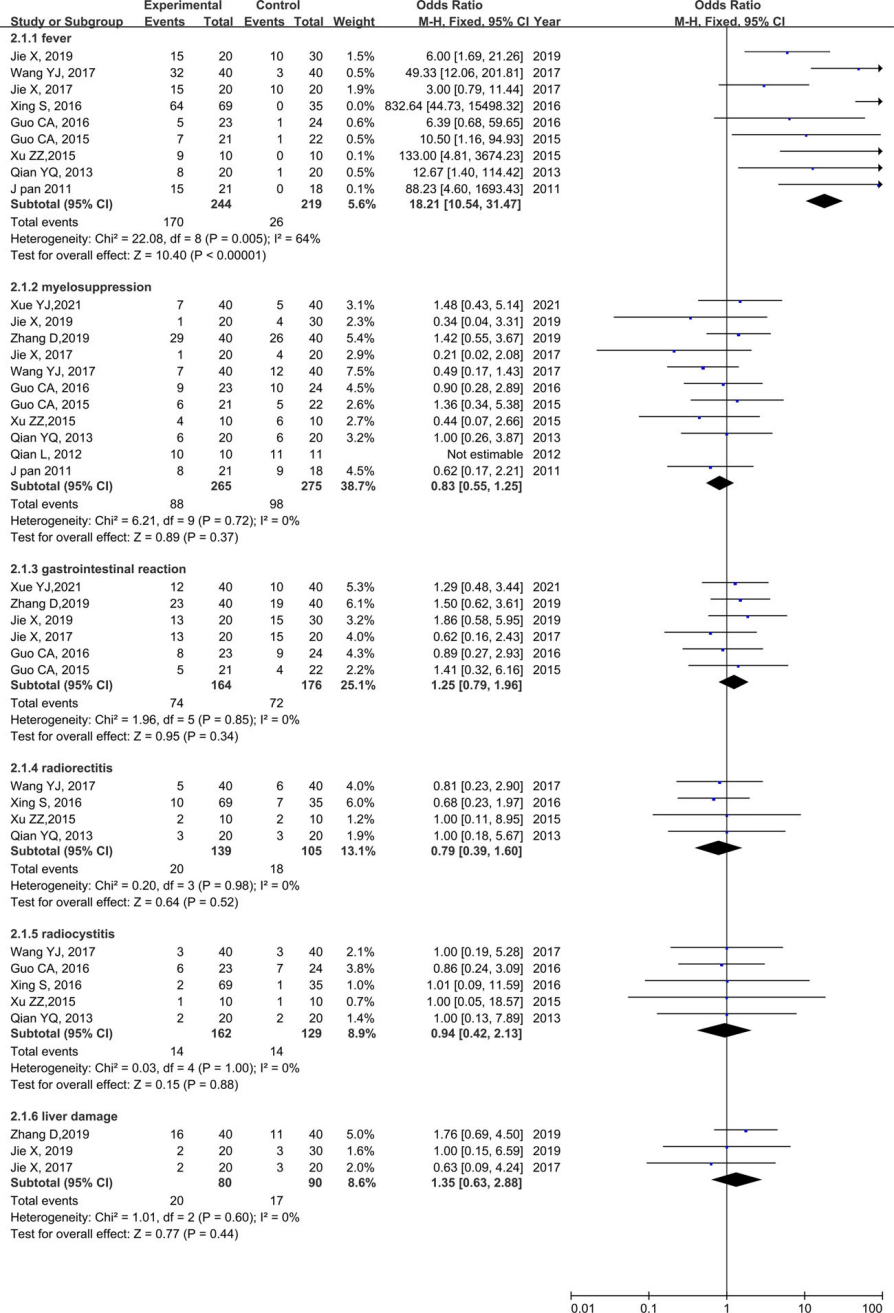
Forest plot for adverse reactions of rAd-p53 combined group and control group.

No heterogeneity was found for any adverse reaction; therefore, a fixed-effects model was used for the analysis. Nine studies reported fever rates (20, 21, 23–27, 29, 30), 11 studies reported rates of myelosuppression (18–21, 23, 25–27, 29–31), and 6 studies reported the rates of gastrointestinal reaction (18–21, 29, 30). Four studies reported the rate of radio rectitis (23–25, 31), five studies reported the rate of radio cystitis (23–25, 29, 31), and three studies reported the rate of liver damage (19–21). The results of the meta-analysis showed a higher fever rate in the rAd-p53 group than that in the no rAd-p53 group (OR = 39.78, 95% CI: 17.96–88.11), *p* < 0.00001], while the other adverse reaction incidences showed no significant differences (**Figure 8**).

### Evaluation of the Sensitivity and Publication Bias

**Figure 9:**
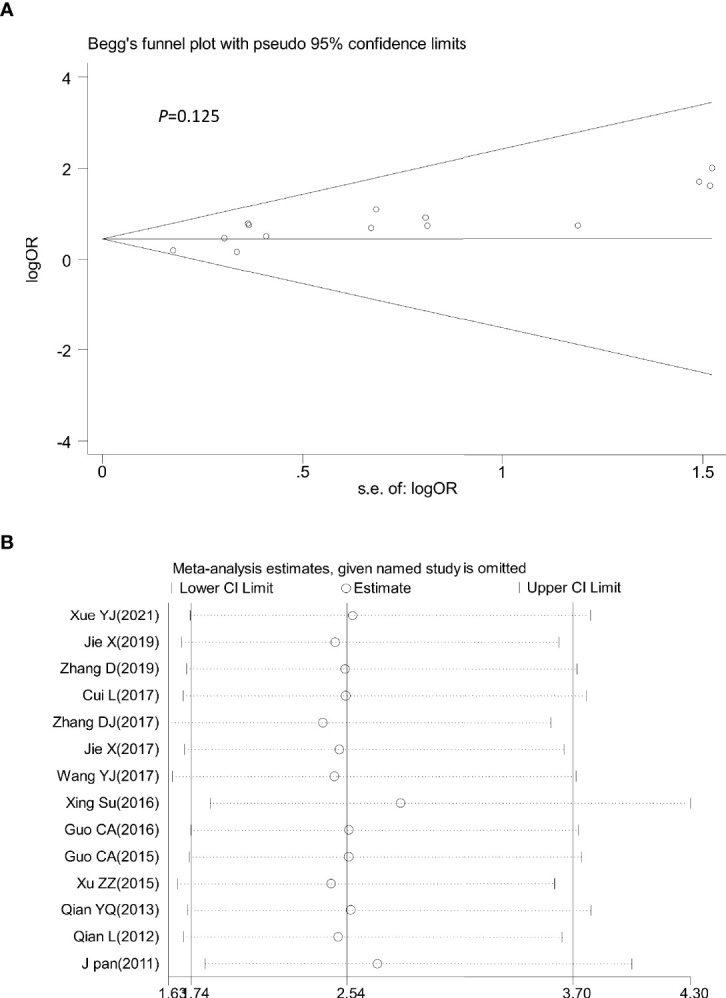
Begg’s funnel plot **(A)** and sensitivity analysis **(B)** of all the included studies for the analysis of CR. Begg’s test (*p* = 0.125). CR, complete remission.

**Figure 10:**
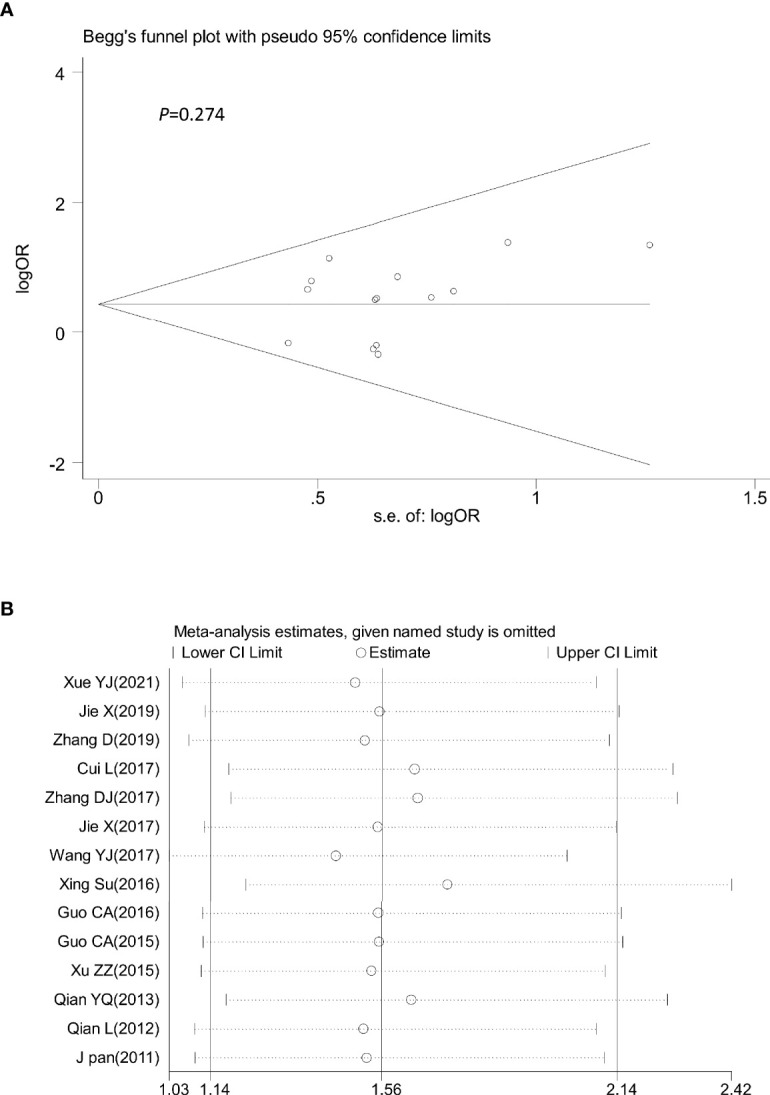
Begg’s funnel plot **(A)** and sensitivity analysis **(B)** of all the included studies for the analysis of PR. Begg’s test (p = 0.274). PR, partial remission.

**Figure 11:**
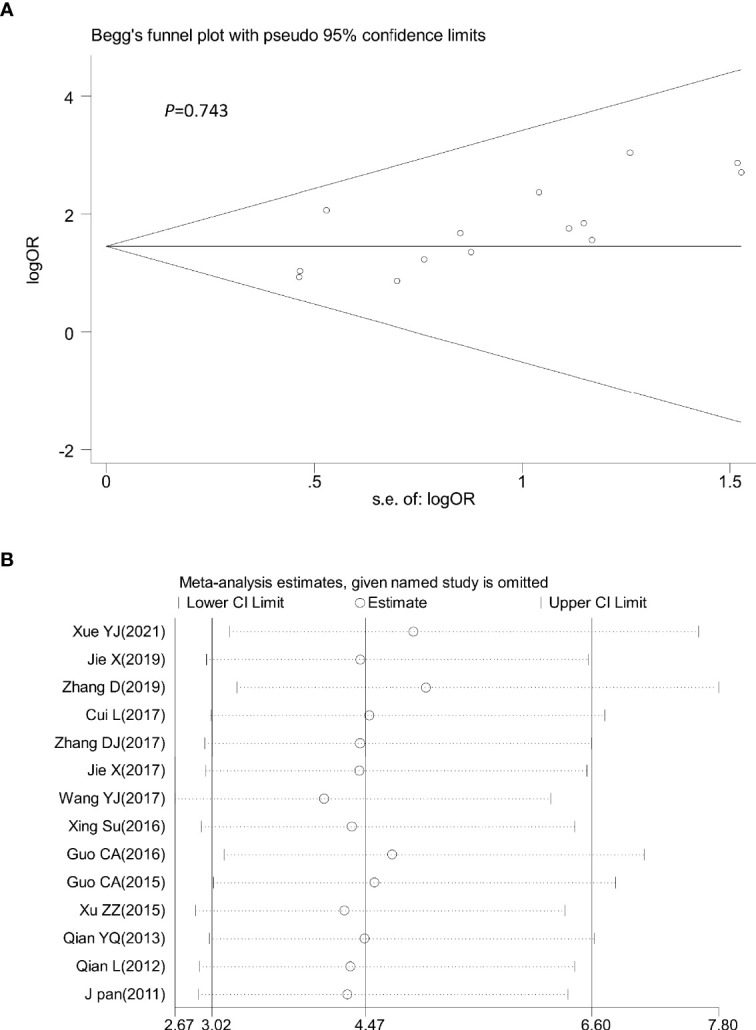
Begg’s funnel plot **(A)** and sensitivity analysis **(B)** of all the included studies for the analysis of ORR. Begg’s test (*p* = 0.743). ORR: objective tumor response.

Sensitivity analysis was performed by omitting one study at a time to assess its influence on the overall estimates. The results showed that the deletion of any single study had no significant effect on the results (**Figures 9B**–**11B**), indicating that the results of this meta-analysis were relatively stable. Analysis of publication bias among the included articles showed no obvious bias in the CR, PR, and ORR. Begg’s funnel plot and Egger’s test indicated no significant publication bias (**Figures 9A**–**11A**, **Supplementary Figures S1–S3**).

## Discussion

RAd-p53 was approved by the State Food and Drug Administration (SFDA) of China in 2004 as a gene therapy medicinal product for tumors. RAd−p53 is a replication-defective living adenovirus carrying the p53 gene. An advantage of the adenovirus delivery system is that it does not result in the integration of the vector DNA into the host cells (32). Once cells have been infected, virus particles cannot replicate but can import the p53 gene, which exerts biological functions. This method has the advantages of high infection efficiency, no genetic toxicity to the human body, and safe clinical application. The basic mechanisms of rhAd−p53 reagents are as follows: (i) the inhibition of tumor growth by cell cycle arrest and induced programmed cell death; (ii) enhancement of chemotherapy−induced cell cycle arrest and apoptosis; (iii) stimulation of the body to produce an anti−tumor immune such that a large number of immune cells gather at the local injection site of tumors; (iv) inhibition of tumor vascular endothelial growth factor (VEGF) to suppress angiogenesis and tumor growth through the “bystander effect”; thus, the injection site of the local tumor tissue will block blood supply and induce tumor necrosis (33).

RAd-p53 is currently used as a treatment for various cancers and has shown remarkable clinical efficacy in head and neck (34), lung (35), liver (36), colorectal (37), and ovarian (38) cancers. Meta-analysis studies have evaluated the safety and efficacy of rAd-p53 for the treatment of nasopharyngeal cancer (39) and malignant pleural effusion (40). To our knowledge, this is the first meta-analysis to systematically assess the effects and safety of rAd-p53 in the treatment of cervical cancer.

This systematic review included 14 studies involving 737 patients. The results of the meta-analysis showed that CR, PR, and ORR in the rAd-p53 combined therapy group were significantly improved compared with the CT/RT/CRT alone group. Similar results were observed in the subgroup analysis. Two of our included 14 studies investigated the long-term survival of rAd-p53 treating cervical cancer. Zhang Dan et al. used the Kaplan–Meier method to estimate the progression‐free survival (PFS) of patients administered rAd-p53 combined with chemotherapy. They reported that the PFS of the experimental group (6.38 ± 0.14) was higher than that of the control group (4.48 ± 0.14). Su et al. showed that the 5-year overall survival rate (OS) for rAd-p53 combined with radiotherapy was 17.5% higher than that for RT alone (hazard ratio [HR] = 0.551, 95% CI: 0.278–1.095, *p* = 0.084); the 5-year PFS for rAd-p53 combined with radiotherapy was 17.1% higher than that for RT alone (HR = 0.485,95% CI: 0.234–1.006, *p* = 0.0470). DNA aneuploidy is thought to reflect the biological behavior of malignancy, in which the higher the DNA aneuploidy of tumor tissue, the higher the degree of malignancy and the worse the prognosis (41). Two of the 14 included articles reported differences in DNA ploidy index before and after rAd-p53 combined with RT for cervical cancer. The results showed no significant difference in DNA aneuploidy between the two groups before treatment. After treatment, the positivity rate of DNA aneuploidy in the rAd-p53 combined group was lower than that in the RT group. The intratumoral injection of rhAd-p53 inhibited VEGF expression and angiogenesis and promoted tumor necrosis and shrinkage in advanced cancer (42). One of the included 14 studies was a related experiment. Xiao et al. determined the expression levels of vascular endothelial VEGF in three sets of cervical cancer specimens (blank control; CT alone; and rAd-p53+CT) before and after treatment by immunohistochemistry. The results showed significantly decreased VEGF expression in the CT alone and rAd-p53+CT groups compared to that in the control. In addition, the rhAd-p53 + CT group showed a more significant decrease in VEGF levels than that in the CT group. Based on the results of the above analyses, rad-p53 may be beneficial in patients with cervical cancer in terms of both short- and long-term effects.

Regarding the incidence of adverse reactions, only the occurrence rate of fever in the rAd-p53 combination group was higher than that in the control group. No significant differences were observed between the groups in myelosuppression, gastrointestinal reaction, radio rectitis, radio cystitis, and liver damage rate. All fevers were transient and self-limited and appeared within 24 h after injection. The grade II fevers resolved spontaneously, while the grade III fevers returned to normal levels after the administration of antipyretics. It is well known that adenovirus vectors can induce a strong immune response in patients, which manifests as a self-limiting fever. Although fever is considered a side effect in clinical use, it also reflects the effectiveness and benefits of Adp53 in mobilizing the immune system. Thus, rAd-p53 was a safe and biologically active treatment for improving the curative effect in patients with cervical cancer.

### Limitations

Some questions remain in our study. The issues requiring further study include the identification of the best chemotherapy regimen to be used in combination with rAd-p53, the recommended dose for radiotherapy, and whether the combination of rAd-p53 as a radiotherapy and chemotherapy sensitizer can produce long-term improvement in distant control. Moreover, most of the included studies did not perform long-term follow-up. Additional studies are needed to evaluate the relationship between rAd-p53 expression and long-term survival.

## Conclusion

In conclusion, our results demonstrated that rAd-p53 combined with CT/RT/CRT was more effective and safer for the treatment of cervical cancer. Therefore, rAd-p53 showed great potential as an effective therapy for cervical cancer.

## Data Availability Statement

The original contributions presented in the study are included in the article/**Supplementary Material**. Further inquiries can be directed to the corresponding authors.

## Author Contributions

YG and JC proposed the study concept and design. YG and XZ collected the literature. YG, MF, MX, and YX analyzed the data. All authors contributed to the article and approved the final manuscript.

## Funding

This work was supported by the National Natural Science Foundation of China (grant no. 81972845), the Research Foundation of Xuzhou Medical University (grant no. D2019033), the Social Development Projects of Xuzhou (KC19144), the Jiangsu Distinguished Professorship Program, the Jiangsu Distinguished Medical Experts program, and the Xuzhou Jinlonghu Distinguished Talents Program.

## Conflict of Interest

The authors declare that the research was conducted in the absence of any commercial or financial relationships that could be construed as a potential conflict of interest.

## Publisher’s Note

All claims expressed in this article are solely those of the authors and do not necessarily represent those of their affiliated organizations, or those of the publisher, the editors and the reviewers. Any product that may be evaluated in this article, or claim that may be made by its manufacturer, is not guaranteed or endorsed by the publisher.
