# Supplementary material for: Recombinant Human Adenovirus-p53 Therapy for the Treatment of Cervical Cancer: A Meta-Analysis
Source: Front Oncol. 2021 Oct 18;11:748681. doi: 10.3389/fonc.2021.748681 (PMC8558497; doi:10.3389/fonc.2021.748681)
Supplement: Supplementary file 1 [file DataSheet_1.docx]

Supplementary Material

# Supplementary materials caption

**TABLE S1** | Summary of the meta-analysis

**Figure S1** | Publication bias of the recruited literatures. Funnel plot of the analysis of CR(**A**),PR(**B**) and ORR(**C**).

**Figure S2** | Egger’s plot of the analysis of publication bias of CR(**A**),PR(**B**) and ORR(**C**).

**Figure S3** | Begg’s and Egger’s tests for publication bias of CR(**A**),PR(**B**) and ORR(**C**).

**Figure S4 |** Forest plot for subgroup analysis of CR(**A**),PR(**B**),ORR(**C**) based on IMRT/Non-IMRT

**Appendix S1** | Pubmed literature search strategies.

| Outcomes and toxicity | | Included study | Number of EXP | Number of CON | Heterogeneity | | Meta-analysis model | Result of meta-analysis | |
| --- | --- | --- | --- | --- | --- | --- | --- | --- | --- |
|  |  |  |  |  | P | I^2^ |  | OR(95%CI) | P |
| CR | all | 14 | 379 | 358 | 0.79 | 0% | fixed-effects | 2.54(1.74-3.70) | <0.00001 |
| subgroup | CT | 4 | 120 | 130 | 0.86 | 0% | fixed-effects | 3.08(1.16-8.14) | 0.02 |
|  | RT | 6 | 181 | 148 | 0.67 | 0% | fixed-effects | 2.37(1.50-3.76) | 0.0002 |
|  | CRT | 4 | 78 | 80 | 0.94 | 0% | fixed-effects | 2.72(1.13-6.52) | 0.02 |
|  | IMRT | 6 | 128 | 133 | 0.96 | 0% | fixed-effects | 3.35(1.86-6.00) | 0.05 |
|  | Non-IMRT | 4 | 131 | 95 | 0.93 | 0% | fixed-effects | 1.79(1.00-3.18) | 0.05 |
| PR | all | 14 | 379 | 358 | 0.67 | 0% | fixed-effects | 1.56(1.14-2.14) | 0.006 |
| subgroup | CT | 4 | 120 | 130 | 0.99 | 0% | fixed-effects | 2.00(1.13-3.52) | 0.0002 |
|  | RT | 6 | 171 | 148 | 0.24 | 26% | fixed-effects | 1.34(0.84-2.14) | 0.22 |
|  | CRT | 4 | 78 | 80 | 0.51 | 0% | fixed-effects | 1.50(0.78-2.89) | 0.22 |
|  | IMRT | 6 | 128 | 133 | 0.30 | 17% | fixed-effects | 1.65(0.98-2.81) | 0.06 |
|  | Non-IMRT | 4 | 131 | 95 | 0.52 | 0% | fixed-effects | 1.16(0.67-2.0) | 0.61 |
| ORR | all | 14 | 379 | 358 | 0.82 | 0% | fixed-effects | 4.47(3.02-6.60) | <0.00001 |
| subgroup | CT | 4 | 120 | 130 | 0.82 | 0% | fixed-effects | 3.10(1.72-5.59) | 0.0002 |
|  | RT | 6 | 171 | 148 | 0.92 | 0% | fixed-effects | 8.43(4.07-17.47) | <0.00001 |
|  | CRT | 4 | 78 | 80 | 0.69 | 0% | fixed-effects | 3.72(1.68-8.21) | 0.001 |
|  | IMRT | 6 | 128 | 133 | 0.61 | 0% | fixed-effects | 5.80(3.13-10.74) | <0.00001 |
|  | Non-IMRT | 4 | 131 | 95 | 0.69 | 0% | fixed-effects | 6.15(2.17-17.45) | 0.0006 |
| Toxicity |  |  |  |  |  |  |  |  |  |
|  | fever | 9 | 244 | 219 | 0.005 | 64% | fixed-effects | 18.21(10.54-31.47) | <0.00001 |
|  | myelosuppression | 11 | 265 | 275 | 0.72 | 0% | fixed-effects | 0.83(0.55-1.25) | 0.37 |
|  | gastrointestinal reaction | 6 | 164 | 176 | 0.85 | 0% | fixed-effects | 1.25(0.79-1.96) | 0.34 |
|  | radiorectitis | 4 | 139 | 105 | 0.98 | 0% | fixed-effects | 0.79(0.39-1.60) | 0.52 |
|  | radiocystitis | 5 | 162 | 129 | 1.00 | 0% | fixed-effects | 0.94(0.42-2.13) | 0.88 |
|  | liver damage | 3 | 80 | 90 | 0.60 | 0% | fixed-effects | 1.35(0.63-2.88) | 0.44 |

**TABLE S1**

## Figure S1

A B

C

## Figure S2

A B

C

## Figure S3

A B


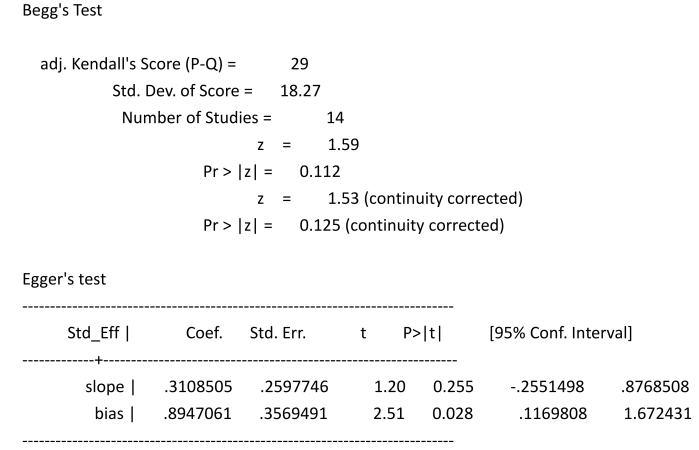

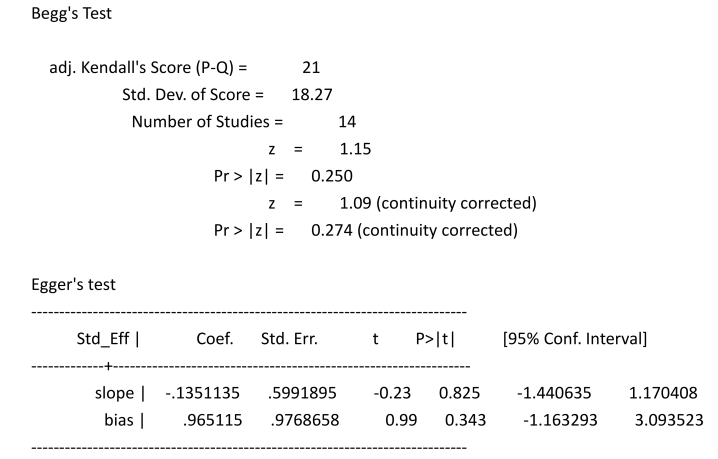


C


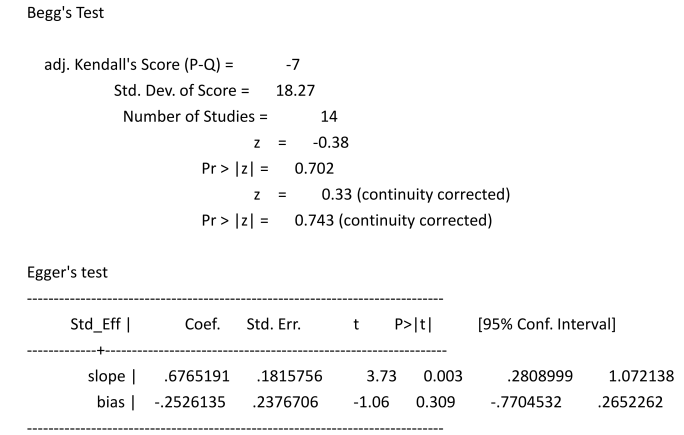


**Figure S4**


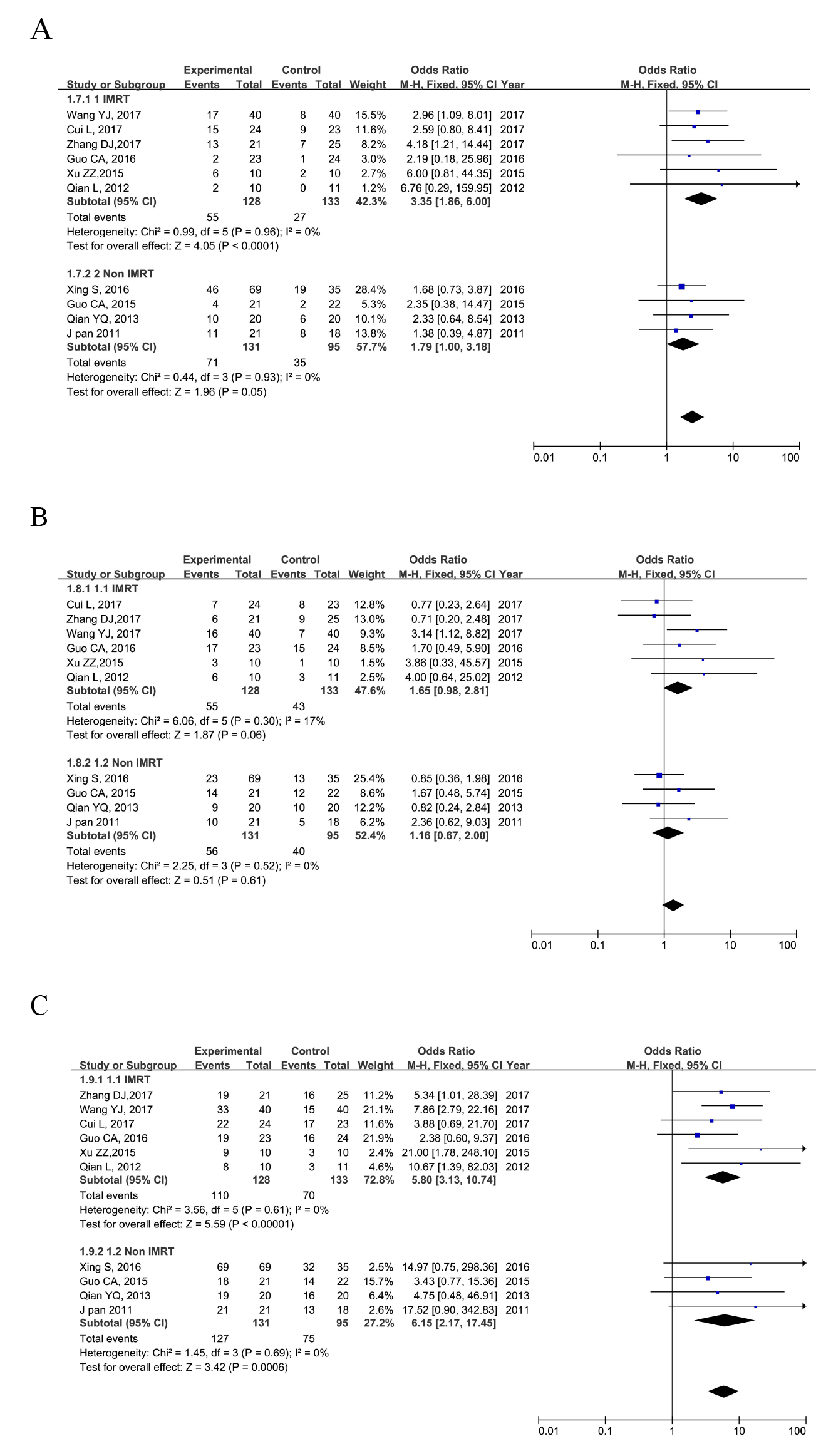


**Appendix S1**

| PubMed Search History | | |
| --- | --- | --- |
| #1 | Search: "Uterine Cervical Neoplasms"[Mesh] | 78046 |
| #2 | Search: ((((((((((((((((((((((((Cervical Neoplasm, Uterine[Title/Abstract]) OR (Cervical Neoplasms, Uterine[Title/Abstract])) OR (Neoplasm, Uterine Cervical[Title/Abstract])) OR (Neoplasms, Uterine Cervical[Title/Abstract])) OR (Uterine Cervical Neoplasm[Title/Abstract])) OR (Neoplasms, Cervical[Title/Abstract])) OR (Cervical Neoplasms[Title/Abstract])) OR (Cervical Neoplasm[Title/Abstract])) OR (Neoplasm, Cervical[Title/Abstract])) OR (Neoplasms, Cervix[Title/Abstract])) OR (Cervix Neoplasms[Title/Abstract])) OR (Cervix Neoplasm[Title/Abstract])) OR (Neoplasm, Cervix[Title/Abstract])) OR (Cancer of the Uterine Cervix[Title/Abstract])) OR (Cancer of the Cervix[Title/Abstract])) OR (Cervical Cancer[Title/Abstract])) OR (Uterine Cervical Cancer[Title/Abstract])) OR (Cancer, Uterine Cervical[Title/Abstract])) OR (Cancers, Uterine Cervical[Title/Abstract])) OR (Cervical Cancer, Uterine[Title/Abstract])) OR (Uterine Cervical Cancers[Title/Abstract])) OR (Cancer of Cervix[Title/Abstract])) OR (Cervix Cancer[Title/Abstract])) OR (Cancer, Cervix[Title/Abstract])) OR (Cancers, Cervix[Title/Abstract]) | 78445 |
| #3 | Search: ("Uterine Cervical Neoplasms"[Mesh]) OR (((((((((((((((((((((((((Cervical Neoplasm, Uterine[Title/Abstract]) OR (Cervical Neoplasms, Uterine[Title/Abstract])) OR (Neoplasm, Uterine Cervical[Title/Abstract])) OR (Neoplasms, Uterine Cervical[Title/Abstract])) OR (Uterine Cervical Neoplasm[Title/Abstract])) OR (Neoplasms, Cervical[Title/Abstract])) OR (Cervical Neoplasms[Title/Abstract])) OR (Cervical Neoplasm[Title/Abstract])) OR (Neoplasm, Cervical[Title/Abstract])) OR (Neoplasms, Cervix[Title/Abstract])) OR (Cervix Neoplasms[Title/Abstract])) OR (Cervix Neoplasm[Title/Abstract])) OR (Neoplasm, Cervix[Title/Abstract])) OR (Cancer of the Uterine Cervix[Title/Abstract])) OR (Cancer of the Cervix[Title/Abstract])) OR (Cervical Cancer[Title/Abstract])) OR (Uterine Cervical Cancer[Title/Abstract])) OR (Cancer, Uterine Cervical[Title/Abstract])) OR (Cancers, Uterine Cervical[Title/Abstract])) OR (Cervical Cancer, Uterine[Title/Abstract])) OR (Uterine Cervical Cancers[Title/Abstract])) OR (Cancer of Cervix[Title/Abstract])) OR (Cervix Cancer[Title/Abstract])) OR (Cancer, Cervix[Title/Abstract])) OR (Cancers, Cervix[Title/Abstract])) | 102215 |
| #4 | Search: ((Recombinant human adenovirus-p53[Title/Abstract]) OR (rAd-p53[Title/Abstract])) OR (Gendicine[Title/Abstract]) | 126 |
| #5 | Search: "Drug Therapy"[Mesh] | 1415218 |
| #6 | Search: ((((((((((((((((((drug therapy[Title/Abstract]) OR (Chemotherapy-Induced Febrile Neutropenia[Title/Abstract])) OR (Consolidation Chemotherapy[Title/Abstract])) OR (Induction Chemotherapy[Title/Abstract])) OR (Maintenance Chemotherapy[Title/Abstract])) OR (Chemotherapy, Adjuvant[Title/Abstract])) OR (Chemotherapy, Cancer, Regional Perfusion[Title/Abstract])) OR (Antineoplastic Combined Chemotherapy Protocols[Title/Abstract])) OR (Hyperthermic Intraperitoneal Chemotherapy[Title/Abstract])) OR (Chemotherapy-Related Cognitive Impairment[Title/Abstract])) OR (Antineoplastic Agents[Title/Abstract])) OR (Hand-Foot Syndrome[Title/Abstract])) OR (Neoadjuvant Therapy[Title/Abstract])) OR (Drug Therapy, Combination[Title/Abstract])) OR (R-CHOP protocol[Title/Abstract])) OR (MAC chemotherapy protocol[Title/Abstract])) OR (EEPFL protocol[Title/Abstract])) OR (ECT chemotherapy protocol[Title/Abstract])) OR (ICE protocol 5[Title/Abstract]) | 81489 |
| #7 | Search: ("Drug Therapy"[Mesh]) OR (((((((((((((((((((drug therapy[Title/Abstract]) OR (Chemotherapy-Induced Febrile Neutropenia[Title/Abstract])) OR (Consolidation Chemotherapy[Title/Abstract])) OR (Induction Chemotherapy[Title/Abstract])) OR (Maintenance Chemotherapy[Title/Abstract])) OR (Chemotherapy, Adjuvant[Title/Abstract])) OR (Chemotherapy, Cancer, Regional Perfusion[Title/Abstract])) OR (Antineoplastic Combined Chemotherapy Protocols[Title/Abstract])) OR (Hyperthermic Intraperitoneal Chemotherapy[Title/Abstract])) OR (Chemotherapy-Related Cognitive Impairment[Title/Abstract])) OR (Antineoplastic Agents[Title/Abstract])) OR (Hand-Foot Syndrome[Title/Abstract])) OR (Neoadjuvant Therapy[Title/Abstract])) OR (Drug Therapy, Combination[Title/Abstract])) OR (R-CHOP protocol[Title/Abstract])) OR (MAC chemotherapy protocol[Title/Abstract])) OR (EEPFL protocol[Title/Abstract])) OR (ECT chemotherapy protocol[Title/Abstract])) OR (ICE protocol 5[Title/Abstract])) | 1464504 |
| #8 | Search: "Radiotherapy"[Mesh] | 193863 |
| #9 | Search: ((((((((((((((((Radiotherapies[Title/Abstract]) OR (Radiation Therapy[Title/Abstract])) OR (Radiation Therapies[Title/Abstract])) OR (Therapies, Radiation[Title/Abstract])) OR (Therapy, Radiation[Title/Abstract])) OR (Radiation Treatment[Title/Abstract])) OR (Radiation Treatments[Title/Abstract])) OR (Treatment, Radiation[Title/Abstract])) OR (Radiotherapy, Targeted[Title/Abstract])) OR (Radiotherapies, Targeted[Title/Abstract])) OR (Targeted Radiotherapies[Title/Abstract])) OR (Targeted Radiotherapy[Title/Abstract])) OR (Targeted Radiation Therapy[Title/Abstract])) OR (Radiation Therapies, Targeted[Title/Abstract])) OR (Targeted Radiation Therapies[Title/Abstract])) OR (Therapies, Targeted Radiation[Title/Abstract])) OR (Therapy, Targeted Radiation[Title/Abstract]) | 91852 |
| #10 | Search: ("Radiotherapy"[Mesh]) OR (((((((((((((((((Radiotherapies[Title/Abstract]) OR (Radiation Therapy[Title/Abstract])) OR (Radiation Therapies[Title/Abstract])) OR (Therapies, Radiation[Title/Abstract])) OR (Therapy, Radiation[Title/Abstract])) OR (Radiation Treatment[Title/Abstract])) OR (Radiation Treatments[Title/Abstract])) OR (Treatment, Radiation[Title/Abstract])) OR (Radiotherapy, Targeted[Title/Abstract])) OR (Radiotherapies, Targeted[Title/Abstract])) OR (Targeted Radiotherapies[Title/Abstract])) OR (Targeted Radiotherapy[Title/Abstract])) OR (Targeted Radiation Therapy[Title/Abstract])) OR (Radiation Therapies, Targeted[Title/Abstract])) OR (Targeted Radiation Therapies[Title/Abstract])) OR (Therapies, Targeted Radiation[Title/Abstract])) OR (Therapy, Targeted Radiation[Title/Abstract])) | 247164 |
| #11 | Search: "Chemoradiotherapy"[Mesh] | 16800 |
| #12 | Search: ((((((((((((((((((((((Chemoradiotherapies[Title/Abstract]) OR (Radiochemotherapy[Title/Abstract])) OR (Radiochemotherapies[Title/Abstract])) OR (Concurrent Chemoradiotherapy[Title/Abstract])) OR (Chemoradiotherapies, Concurrent[Title/Abstract])) OR (Chemoradiotherapy, Concurrent[Title/Abstract])) OR (Concurrent Chemoradiotherapies[Title/Abstract])) OR (Synchronous Chemoradiotherapy[Title/Abstract])) OR (Chemoradiotherapies, Synchronous[Title/Abstract])) OR (Chemoradiotherapy, Synchronous[Title/Abstract])) OR (Synchronous Chemoradiotherapies[Title/Abstract])) OR (Concurrent Radiochemotherapy[Title/Abstract])) OR (Concurrent Radiochemotherapies[Title/Abstract])) OR (Radiochemotherapies, Concurrent[Title/Abstract])) OR (Radiochemotherapy, Concurrent[Title/Abstract])) OR (Concomitant Chemoradiotherapy[Title/Abstract])) OR (Chemoradiotherapies, Concomitant[Title/Abstract])) OR (Chemoradiotherapy, Concomitant[Title/Abstract])) OR (Concomitant Chemoradiotherapies[Title/Abstract])) OR (Concomitant Radiochemotherapy[Title/Abstract])) OR (Concomitant Radiochemotherapies[Title/Abstract])) OR (Radiochemotherapies, Concomitant[Title/Abstract])) OR (Radiochemotherapy, Concomitant[Title/Abstract]) | 14235 |
| #13 | Search: ("Chemoradiotherapy"[Mesh]) OR (((((((((((((((((((((((Chemoradiotherapies[Title/Abstract]) OR (Radiochemotherapy[Title/Abstract])) OR (Radiochemotherapies[Title/Abstract])) OR (Concurrent Chemoradiotherapy[Title/Abstract])) OR (Chemoradiotherapies, Concurrent[Title/Abstract])) OR (Chemoradiotherapy, Concurrent[Title/Abstract])) OR (Concurrent Chemoradiotherapies[Title/Abstract])) OR (Synchronous Chemoradiotherapy[Title/Abstract])) OR (Chemoradiotherapies, Synchronous[Title/Abstract])) OR (Chemoradiotherapy, Synchronous[Title/Abstract])) OR (Synchronous Chemoradiotherapies[Title/Abstract])) OR (Concurrent Radiochemotherapy[Title/Abstract])) OR (Concurrent Radiochemotherapies[Title/Abstract])) OR (Radiochemotherapies, Concurrent[Title/Abstract])) OR (Radiochemotherapy, Concurrent[Title/Abstract])) OR (Concomitant Chemoradiotherapy[Title/Abstract])) OR (Chemoradiotherapies, Concomitant[Title/Abstract])) OR (Chemoradiotherapy, Concomitant[Title/Abstract])) OR (Concomitant Chemoradiotherapies[Title/Abstract])) OR (Concomitant Radiochemotherapy[Title/Abstract])) OR (Concomitant Radiochemotherapies[Title/Abstract])) OR (Radiochemotherapies, Concomitant[Title/Abstract])) OR (Radiochemotherapy, Concomitant[Title/Abstract])) | 25551 |
| #14 | Search: ((("Drug Therapy"[Mesh]) OR (((((((((((((((((((drug therapy[Title/Abstract]) OR (Chemotherapy-Induced Febrile Neutropenia[Title/Abstract])) OR (Consolidation Chemotherapy[Title/Abstract])) OR (Induction Chemotherapy[Title/Abstract])) OR (Maintenance Chemotherapy[Title/Abstract])) OR (Chemotherapy, Adjuvant[Title/Abstract])) OR (Chemotherapy, Cancer, Regional Perfusion[Title/Abstract])) OR (Antineoplastic Combined Chemotherapy Protocols[Title/Abstract])) OR (Hyperthermic Intraperitoneal Chemotherapy[Title/Abstract])) OR (Chemotherapy-Related Cognitive Impairment[Title/Abstract])) OR (Antineoplastic Agents[Title/Abstract])) OR (Hand-Foot Syndrome[Title/Abstract])) OR (Neoadjuvant Therapy[Title/Abstract])) OR (Drug Therapy, Combination[Title/Abstract])) OR (R-CHOP protocol[Title/Abstract])) OR (MAC chemotherapy protocol[Title/Abstract])) OR (EEPFL protocol[Title/Abstract])) OR (ECT chemotherapy protocol[Title/Abstract])) OR (ICE protocol 5[Title/Abstract]))) OR (("Radiotherapy"[Mesh]) OR (((((((((((((((((Radiotherapies[Title/Abstract]) OR (Radiation Therapy[Title/Abstract])) OR (Radiation Therapies[Title/Abstract])) OR (Therapies, Radiation[Title/Abstract])) OR (Therapy, Radiation[Title/Abstract])) OR (Radiation Treatment[Title/Abstract])) OR (Radiation Treatments[Title/Abstract])) OR (Treatment, Radiation[Title/Abstract])) OR (Radiotherapy, Targeted[Title/Abstract])) OR (Radiotherapies, Targeted[Title/Abstract])) OR (Targeted Radiotherapies[Title/Abstract])) OR (Targeted Radiotherapy[Title/Abstract])) OR (Targeted Radiation Therapy[Title/Abstract])) OR (Radiation Therapies, Targeted[Title/Abstract])) OR (Targeted Radiation Therapies[Title/Abstract])) OR (Therapies, Targeted Radiation[Title/Abstract])) OR (Therapy, Targeted Radiation[Title/Abstract])))) OR (("Chemoradiotherapy"[Mesh]) OR (((((((((((((((((((((((Chemoradiotherapies[Title/Abstract]) OR (Radiochemotherapy[Title/Abstract])) OR (Radiochemotherapies[Title/Abstract])) OR (Concurrent Chemoradiotherapy[Title/Abstract])) OR (Chemoradiotherapies, Concurrent[Title/Abstract])) OR (Chemoradiotherapy, Concurrent[Title/Abstract])) OR (Concurrent Chemoradiotherapies[Title/Abstract])) OR (Synchronous Chemoradiotherapy[Title/Abstract])) OR (Chemoradiotherapies, Synchronous[Title/Abstract])) OR (Chemoradiotherapy, Synchronous[Title/Abstract])) OR (Synchronous Chemoradiotherapies[Title/Abstract])) OR (Concurrent Radiochemotherapy[Title/Abstract])) OR (Concurrent Radiochemotherapies[Title/Abstract])) OR (Radiochemotherapies, Concurrent[Title/Abstract])) OR (Radiochemotherapy, Concurrent[Title/Abstract])) OR (Concomitant Chemoradiotherapy[Title/Abstract])) OR (Chemoradiotherapies, Concomitant[Title/Abstract])) OR (Chemoradiotherapy, Concomitant[Title/Abstract])) OR (Concomitant Chemoradiotherapies[Title/Abstract])) OR (Concomitant Radiochemotherapy[Title/Abstract])) OR (Concomitant Radiochemotherapies[Title/Abstract])) OR (Radiochemotherapies, Concomitant[Title/Abstract])) OR (Radiochemotherapy, Concomitant[Title/Abstract]))) | 1667633 |
| #15 | Search: (((Recombinant human adenovirus-p53[Title/Abstract]) OR (rAd-p53[Title/Abstract])) OR (Gendicine[Title/Abstract])) AND (((("Drug Therapy"[Mesh]) OR (((((((((((((((((((drug therapy[Title/Abstract]) OR (Chemotherapy-Induced Febrile Neutropenia[Title/Abstract])) OR (Consolidation Chemotherapy[Title/Abstract])) OR (Induction Chemotherapy[Title/Abstract])) OR (Maintenance Chemotherapy[Title/Abstract])) OR (Chemotherapy, Adjuvant[Title/Abstract])) OR (Chemotherapy, Cancer, Regional Perfusion[Title/Abstract])) OR (Antineoplastic Combined Chemotherapy Protocols[Title/Abstract])) OR (Hyperthermic Intraperitoneal Chemotherapy[Title/Abstract])) OR (Chemotherapy-Related Cognitive Impairment[Title/Abstract])) OR (Antineoplastic Agents[Title/Abstract])) OR (Hand-Foot Syndrome[Title/Abstract])) OR (Neoadjuvant Therapy[Title/Abstract])) OR (Drug Therapy, Combination[Title/Abstract])) OR (R-CHOP protocol[Title/Abstract])) OR (MAC chemotherapy protocol[Title/Abstract])) OR (EEPFL protocol[Title/Abstract])) OR (ECT chemotherapy protocol[Title/Abstract])) OR (ICE protocol 5[Title/Abstract]))) OR (("Radiotherapy"[Mesh]) OR (((((((((((((((((Radiotherapies[Title/Abstract]) OR (Radiation Therapy[Title/Abstract])) OR (Radiation Therapies[Title/Abstract])) OR (Therapies, Radiation[Title/Abstract])) OR (Therapy, Radiation[Title/Abstract])) OR (Radiation Treatment[Title/Abstract])) OR (Radiation Treatments[Title/Abstract])) OR (Treatment, Radiation[Title/Abstract])) OR (Radiotherapy, Targeted[Title/Abstract])) OR (Radiotherapies, Targeted[Title/Abstract])) OR (Targeted Radiotherapies[Title/Abstract])) OR (Targeted Radiotherapy[Title/Abstract])) OR (Targeted Radiation Therapy[Title/Abstract])) OR (Radiation Therapies, Targeted[Title/Abstract])) OR (Targeted Radiation Therapies[Title/Abstract])) OR (Therapies, Targeted Radiation[Title/Abstract])) OR (Therapy, Targeted Radiation[Title/Abstract])))) OR (("Chemoradiotherapy"[Mesh]) OR (((((((((((((((((((((((Chemoradiotherapies[Title/Abstract]) OR (Radiochemotherapy[Title/Abstract])) OR (Radiochemotherapies[Title/Abstract])) OR (Concurrent Chemoradiotherapy[Title/Abstract])) OR (Chemoradiotherapies, Concurrent[Title/Abstract])) OR (Chemoradiotherapy, Concurrent[Title/Abstract])) OR (Concurrent Chemoradiotherapies[Title/Abstract])) OR (Synchronous Chemoradiotherapy[Title/Abstract])) OR (Chemoradiotherapies, Synchronous[Title/Abstract])) OR (Chemoradiotherapy, Synchronous[Title/Abstract])) OR (Synchronous Chemoradiotherapies[Title/Abstract])) OR (Concurrent Radiochemotherapy[Title/Abstract])) OR (Concurrent Radiochemotherapies[Title/Abstract])) OR (Radiochemotherapies, Concurrent[Title/Abstract])) OR (Radiochemotherapy, Concurrent[Title/Abstract])) OR (Concomitant Chemoradiotherapy[Title/Abstract])) OR (Chemoradiotherapies, Concomitant[Title/Abstract])) OR (Chemoradiotherapy, Concomitant[Title/Abstract])) OR (Concomitant Chemoradiotherapies[Title/Abstract])) OR (Concomitant Radiochemotherapy[Title/Abstract])) OR (Concomitant Radiochemotherapies[Title/Abstract])) OR (Radiochemotherapies, Concomitant[Title/Abstract])) OR (Radiochemotherapy, Concomitant[Title/Abstract])))) | 29 |
| #16 | Search: randomized controlled trial[Publication Type] OR randomized[Title/Abstract] OR placebo[Title/Abstract] | 902398 |
| #17 | Search: ((("Uterine Cervical Neoplasms"[Mesh]) OR (((((((((((((((((((((((((Cervical Neoplasm, Uterine[Title/Abstract]) OR (Cervical Neoplasms, Uterine[Title/Abstract])) OR (Neoplasm, Uterine Cervical[Title/Abstract])) OR (Neoplasms, Uterine Cervical[Title/Abstract])) OR (Uterine Cervical Neoplasm[Title/Abstract])) OR (Neoplasms, Cervical[Title/Abstract])) OR (Cervical Neoplasms[Title/Abstract])) OR (Cervical Neoplasm[Title/Abstract])) OR (Neoplasm, Cervical[Title/Abstract])) OR (Neoplasms, Cervix[Title/Abstract])) OR (Cervix Neoplasms[Title/Abstract])) OR (Cervix Neoplasm[Title/Abstract])) OR (Neoplasm, Cervix[Title/Abstract])) OR (Cancer of the Uterine Cervix[Title/Abstract])) OR (Cancer of the Cervix[Title/Abstract])) OR (Cervical Cancer[Title/Abstract])) OR (Uterine Cervical Cancer[Title/Abstract])) OR (Cancer, Uterine Cervical[Title/Abstract])) OR (Cancers, Uterine Cervical[Title/Abstract])) OR (Cervical Cancer, Uterine[Title/Abstract])) OR (Uterine Cervical Cancers[Title/Abstract])) OR (Cancer of Cervix[Title/Abstract])) OR (Cervix Cancer[Title/Abstract])) OR (Cancer, Cervix[Title/Abstract])) OR (Cancers, Cervix[Title/Abstract]))) AND ((((Recombinant human adenovirus-p53[Title/Abstract]) OR (rAd-p53[Title/Abstract])) OR (Gendicine[Title/Abstract])) AND (((("Drug Therapy"[Mesh]) OR (((((((((((((((((((drug therapy[Title/Abstract]) OR (Chemotherapy-Induced Febrile Neutropenia[Title/Abstract])) OR (Consolidation Chemotherapy[Title/Abstract])) OR (Induction Chemotherapy[Title/Abstract])) OR (Maintenance Chemotherapy[Title/Abstract])) OR (Chemotherapy, Adjuvant[Title/Abstract])) OR (Chemotherapy, Cancer, Regional Perfusion[Title/Abstract])) OR (Antineoplastic Combined Chemotherapy Protocols[Title/Abstract])) OR (Hyperthermic Intraperitoneal Chemotherapy[Title/Abstract])) OR (Chemotherapy-Related Cognitive Impairment[Title/Abstract])) OR (Antineoplastic Agents[Title/Abstract])) OR (Hand-Foot Syndrome[Title/Abstract])) OR (Neoadjuvant Therapy[Title/Abstract])) OR (Drug Therapy, Combination[Title/Abstract])) OR (R-CHOP protocol[Title/Abstract])) OR (MAC chemotherapy protocol[Title/Abstract])) OR (EEPFL protocol[Title/Abstract])) OR (ECT chemotherapy protocol[Title/Abstract])) OR (ICE protocol 5[Title/Abstract]))) OR (("Radiotherapy"[Mesh]) OR (((((((((((((((((Radiotherapies[Title/Abstract]) OR (Radiation Therapy[Title/Abstract])) OR (Radiation Therapies[Title/Abstract])) OR (Therapies, Radiation[Title/Abstract])) OR (Therapy, Radiation[Title/Abstract])) OR (Radiation Treatment[Title/Abstract])) OR (Radiation Treatments[Title/Abstract])) OR (Treatment, Radiation[Title/Abstract])) OR (Radiotherapy, Targeted[Title/Abstract])) OR (Radiotherapies, Targeted[Title/Abstract])) OR (Targeted Radiotherapies[Title/Abstract])) OR (Targeted Radiotherapy[Title/Abstract])) OR (Targeted Radiation Therapy[Title/Abstract])) OR (Radiation Therapies, Targeted[Title/Abstract])) OR (Targeted Radiation Therapies[Title/Abstract])) OR (Therapies, Targeted Radiation[Title/Abstract])) OR (Therapy, Targeted Radiation[Title/Abstract])))) OR (("Chemoradiotherapy"[Mesh]) OR (((((((((((((((((((((((Chemoradiotherapies[Title/Abstract]) OR (Radiochemotherapy[Title/Abstract])) OR (Radiochemotherapies[Title/Abstract])) OR (Concurrent Chemoradiotherapy[Title/Abstract])) OR (Chemoradiotherapies, Concurrent[Title/Abstract])) OR (Chemoradiotherapy, Concurrent[Title/Abstract])) OR (Concurrent Chemoradiotherapies[Title/Abstract])) OR (Synchronous Chemoradiotherapy[Title/Abstract])) OR (Chemoradiotherapies, Synchronous[Title/Abstract])) OR (Chemoradiotherapy, Synchronous[Title/Abstract])) OR (Synchronous Chemoradiotherapies[Title/Abstract])) OR (Concurrent Radiochemotherapy[Title/Abstract])) OR (Concurrent Radiochemotherapies[Title/Abstract])) OR (Radiochemotherapies, Concurrent[Title/Abstract])) OR (Radiochemotherapy, Concurrent[Title/Abstract])) OR (Concomitant Chemoradiotherapy[Title/Abstract])) OR (Chemoradiotherapies, Concomitant[Title/Abstract])) OR (Chemoradiotherapy, Concomitant[Title/Abstract])) OR (Concomitant Chemoradiotherapies[Title/Abstract])) OR (Concomitant Radiochemotherapy[Title/Abstract])) OR (Concomitant Radiochemotherapies[Title/Abstract])) OR (Radiochemotherapies, Concomitant[Title/Abstract])) OR (Radiochemotherapy, Concomitant[Title/Abstract])))))) AND (randomized controlled trial[Publication Type] OR randomized[Title/Abstract] OR placebo[Title/Abstract]) | 3 |

**
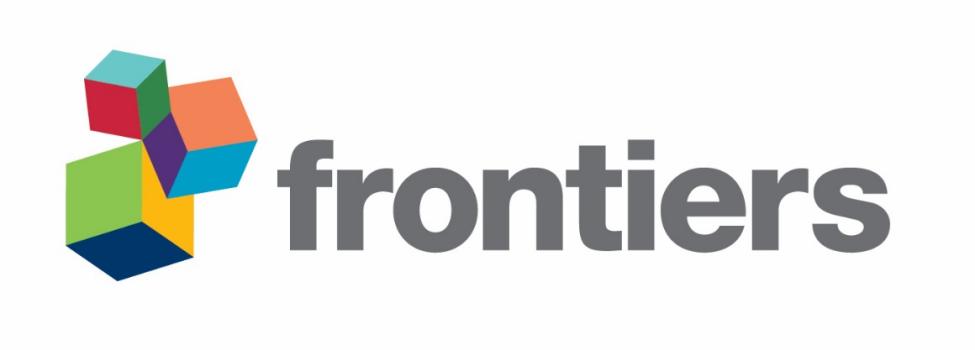
**
